# The Axonal Guidance Receptor Neogenin Promotes Acute Inflammation

**DOI:** 10.1371/journal.pone.0032145

**Published:** 2012-03-06

**Authors:** Klemens König, Dimitra Gatidou, Tiago Granja, Jens Meier, Peter Rosenberger, Valbona Mirakaj

**Affiliations:** 1 Department of Anesthesiology and Intensive Care Medicine, Tübingen University Hospital, Eberhard-Karls University Tübingen, Tübingen, Germany; 2 Clinic of Anesthesiology, Intensive Care Medicine and Pain Therapy, University Hospital Frankfurt am Main, Johann Wolfgang Goethe University, Frankfurt am Main, Germany; University of Colorado Denver, United States of America

## Abstract

Neuronal guidance proteins (NGP) were originally described in the context of axonal growth and migration. Yet recent work has demonstrated that NGPs also serve as guidance cues for immune competent cells. A crucial target receptor for NGPs during embryonic development is the neogenin receptor, however its role during acute inflammation is unknown. We report here that neogenin is abundantly expressed outside the nervous system and that animals with endogenous repression of neogenin (*Neo1^−/−^*) demonstrate attenuated changes of acute inflammation. Studies using functional inhibition of neogenin resulted in a significant attenuation of inflammatory peritonitis. In studies employing bone marrow chimeric animals we found the hematopoietic presence of *Neo1^−/−^* to be responsible for the attenuated inflammatory response. Taken together our studies suggest that the guidance receptor neogenin holds crucial importance for the propagation of an acute inflammatory response and further define mechanisms shared between the nervous and the immune system.

## Introduction

Inflammatory conditions such as sepsis are marked by acute inflammatory changes within the affected tissue sites. During this inflammatory response immune competent cells extravasate from the vascular space and migrate along a chemotactic gradient created by chemokines to reach the site of inflammation [1], [2]. Recent work has identified a group of guidance cues originally identified in the context of nervous system development and described their influence on leukocyte migration and anti-inflammatory potential [3], [4]. To date, several families of these neuronal guidance proteins (NGP)s were described outside the central nervous system (CNS), amongst these are the slits, semaphorins, netrins and the repulsive guidance protein family, where they act as guidance cues for the migration of granulocytes, lymphocytes, monocytes and dendritic cells [5]–[11].

An important target receptor to mediate NGP function in the nervous system is the neogenin receptor, a type I transmembrane protein that significantly influences axonal guidance and neuronal survival. Neogenin is expressed on maturing neuronal populations, including those in the developing cortex, midbrain and hindbrain [12], [13]. The function of neogenin during axonal growth is dependent on the ligand binding to it. Upon binding of netrin-1 neogenin acts as a chemoattractive receptor that activates neuronal migration but in contrast, when binding to the repulsive guidance molecule A (RGM-A) neogenin serves as a chemorepulsive receptor [14], [15]. Furthermore, in the absence of RGM-A neogenin induces cell death in the neuronal tube through activation of caspase 3, whereas in the presence of RGM-A caspase 3 is inhibited resulting in an inhibition of apoptosis [16], [17]. Neogenin is also involved into the development of the embryonic tissue of the mammary gland, into chondrogenesis and angiogenesis [18]–[20]. Park et al. demonstrated that netrin-1 binding to neogenin promotes the migration of smooth muscle cells during angiogenesis [21]. We recently identified that RGM-A dampens an acute inflammatory response following acute peritonitis in dependence of the neogenin receptor [11]. A role for RGM-A was also described during multiple sclerosis by Muramatsu et al. In this study the authors demonstrated that exposure of RGM-A to CD4(+) T cells led to activation of the small GTPase Rap1 and increased adhesion of T cells to intracellular adhesion molecule-1 [5]. This implies that neogenin holds an intrinsic role during inflammatory events that is not known to date.

Based on the fact that NGPs hold important function outside the CNS we pursued the role of neogenin during an acute inflammatory response. We found that animals with endogenous repression of neogenin (*Neo1^−/−^*) demonstrated decreased inflammatory changes in the peritoneal cavity following zymosan A (ZyA) induced peritonits. Functional inhibition of neogenin resulted in a significant attenuation of this inflammatory response. Studies employing chimeric animals identified an important role of hematopoietic neogenin for the promotion of an acute inflammatory response.

## Methods

### *Neogenin^−/−^* (*Neo1^−/−^*) animals

*Neo1^−/−^* receptor knock-out mice were kindly provided by Dr. Tessier-Lavigne [22].

### Peritonitis Model

All animal protocols were in accordance with the German guidelines for use of living animals and were approved by the Institutional Animal Care and Use Committee of the Tübingen University Hospital and the Regierungspräsidium Tübingen. Mice were injected i.p with either 1 ml of NaCl 0.9% or 1 ml NaCl 0.9% containing zymosan A (ZyA, 1 mg/ml, Sigma Chemicals) to induce peritonitis. In a subset of experiments animals were injected with either functionally inhibiting anti-Neo1 antibody (1 µg in 150 µl, R&D Systems, AF 1079) or 1 µg IgG control antibody. Recruited leukocytes were obtained 8 hours later by peritoneal lavage with calcium- and magnesium-free ice-cold PBS solution (5 ml) containing 10 U/ml unfractionated heparin. Collected cells were washed, resuspended in 2 ml of Hanks' balanced salt solution, counted and cytospin samples were prepared. All reagents used were endotoxin-free.

### Transcriptional Analysis

Murine transcriptional analysis of Neogenin mRNA levels was performed using sense primer 5′-GCT GCT CTC ACA GTC AAT GG -3 and 5- GCA TAA CCT CGG ACC ACA AT -3 antisense primer. Murine β -actin expression was evaluated with: sense 5′-CTC TCC CTC ACG CCA TCC TG-3′ and antisense 5′-TCA CGC ACG ATT TCC CTC TCA G-3′.

### Protein analysis

For Western blot analysis animal samples were homogenized, normalized for protein levels and applied to SDS containing polyacrylamid gels. Antibodies used for Western blotting included monoclonal anti–Neogenin (anti-Neo1; sc 15337, Santa Cruz Biotechnology) for murine neogenin analysis. Actin was stained using monoclonal rabbit anti- Actin (Cell Signaling). Blots were washed, and species-matched peroxidase-conjugated secondary antibody (Santa Cruz Biotechnoloy) was added. Labeled bands from washed blots were detected by enhanced chemiluminescence (Amersham Pharmacia Biotech).

### Flow-cytometry

Blood was collected, pooled and incubated with erythrocytes lysing solution for 5 min at room temperature. Cells were then incubated with PE conjugated rabbit anti-Neo1 (Santa Cruz 15337), PerCp conjugated anti-CD 45 (BD Bioscience 5572) or isotype matched control antibody. Flow-cytometry was performed using BD FACS Canto™ II employing BD FACS Diva™ Software.

### Histopathological evaluation

Following peritonitis, mice were killed and tissues fixed in 3,75% formaldehyde solution. Tissues were then embedded in paraffin and stained with H&E. Pictures of tissue sections were taken using Leica Microscope (DM-RBE).

### Generation of Chimeric Animals

To define the contribution of the myeloid and tissue specific Neo1 we generated bone marrow chimeric mice as described previously [6], [23]. Briefly, male donor mice (8–10 weeks old, 20–25 g) were euthanized and the marrow from the tibia and femur was harvested by flushing the marrow cavity with sterile isotonic sodium chloride solution. The bone marrow cells were then centrifuged at 400×g for 5 minutes, resuspended and counted. Recipient mice (8–10 weeks of age, 20–25 g) were irradiated with a total dose of 12 Gy from a ^137^Cs source. Immediately after irradiation, 10^7^ BM cells/recipient were injected in 0.2 ml 0.9% sodium chloride into the jugular vein. Bone marrow was transferred from characterized *Neo1* ***^−/−^*** mice to WT animals and *vice versa*. To control for non specific radiation effects bone marrow was transplanted from WT→WT and *Neo1* ***^−/−^***→*Neo1* ***^−/−^***mice. Success of transplantation was controlled by identifying switch of hematopoetic Neo1 expression through real time PCR and Western blot analysis.

### Measurement of serum cytokines

Tumor necrosis factor α (TNF- α), interleukin (IL)-1β, IL-6, and KC were measured in the peritoneal lavage of experimental animals by standard ELISA (R&D Systems).

### Data analysis

All values are expressed as mean ± SEM. Using Kolmogorov-Smirnov test we could show that the measured values were approximately normally distributed. Statistical significance was determined using one-way ANOVA followed by Bonferroni's multiple-comparison test. Student's *t* test was used where appropriate. A value of *P*<0.05 was considered significant.

## Results

### Neogenin is expressed in murine tissues ouside the CNS

**Figure 1:**
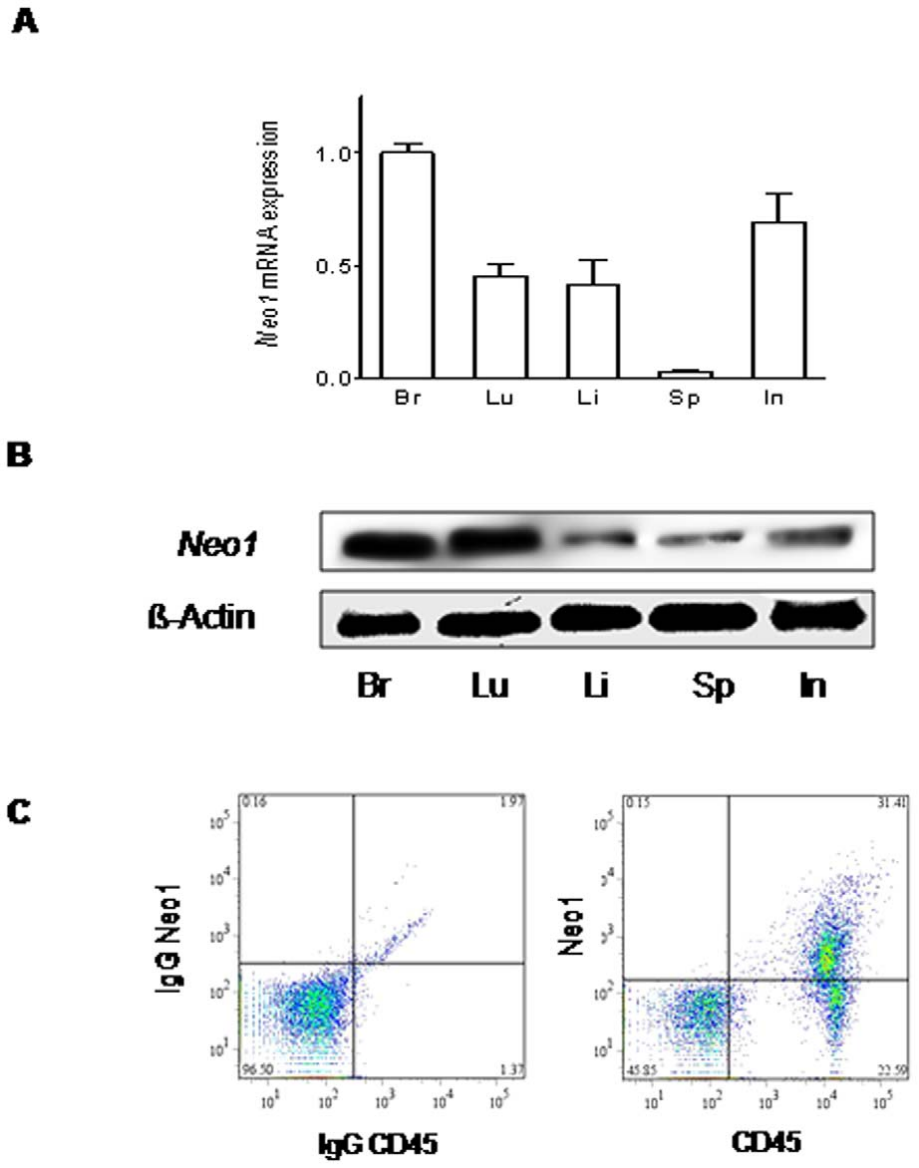
Neogenin is expressed outside the CNS. **A**) Neogenin (*Neo 1*) mRNA expression in the brain (Br), lung (Lu), liver (Li), spleen (Sp) and intestine (In) of WT animals **B**) Pooled Western blot analysis of four independent animals demonstrating *Neo1* expression in (Br), lung (Lu), liver (Li), spleen (Sp) and intestine (In) **C**) Flow-cytometry of murine blood stained with anti-Neo1, anti-CD45 or isotype-matched control antibody (Data are Mean ± SEM, n = 4 per condition).

We initially addressed the question whether Neo1 is expressed in murine organs outside the central nervous system to validate our model subsequently used. We found robust mRNa and protein expression of Neo1 within several murine organs (Figure 1A and B). To confirm the presence of Neo1 on immune competent cells, we next performed flow-cytometry for Neo1. Using CD45 as a general marker for leukocytes we found a robust signal for Neo1 within murine whole blood (Figure 1C).

### Neogenin enhances acute inflammatory peritonitis

**Figure 2:**
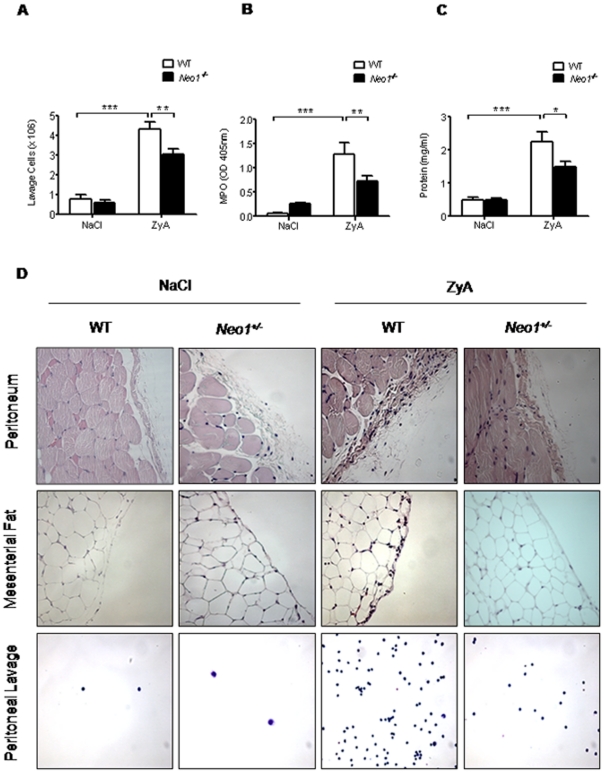
Neogenin enhances acute inflammatory peritonitis. *Neo1^−/−^* and WT animals were injected i.p. with NaCl or 1 mg of zymosan A (ZyA) and peritoneal lavage obtained after 8 hours **A**) Cell count in peritoneal lavage of *Neo1^−/−^* and WT animals **B**) Myeloperoxidase (MPO) activity in peritoneal lavage **C**) Protein content in peritoneal lavage of *Neo1^−/−^* and WT animals **D**) Representative histological analysis of the peritoneum, the mesenterial fat and cytospin samples of the peritoneal lavage in *Neo 1^−/−^* and WT animals 8 hours following intraperitoneal NaCl 0.9% or ZyA injection. Sections prepared with hematoxylin-eosin staining (Magnification ×400, insert ×1000; Data are Mean ± SEM, n = 6 per group, *P<0.05, **P<0.01, ***P<0.001 as indicated).

**Figure 3:**
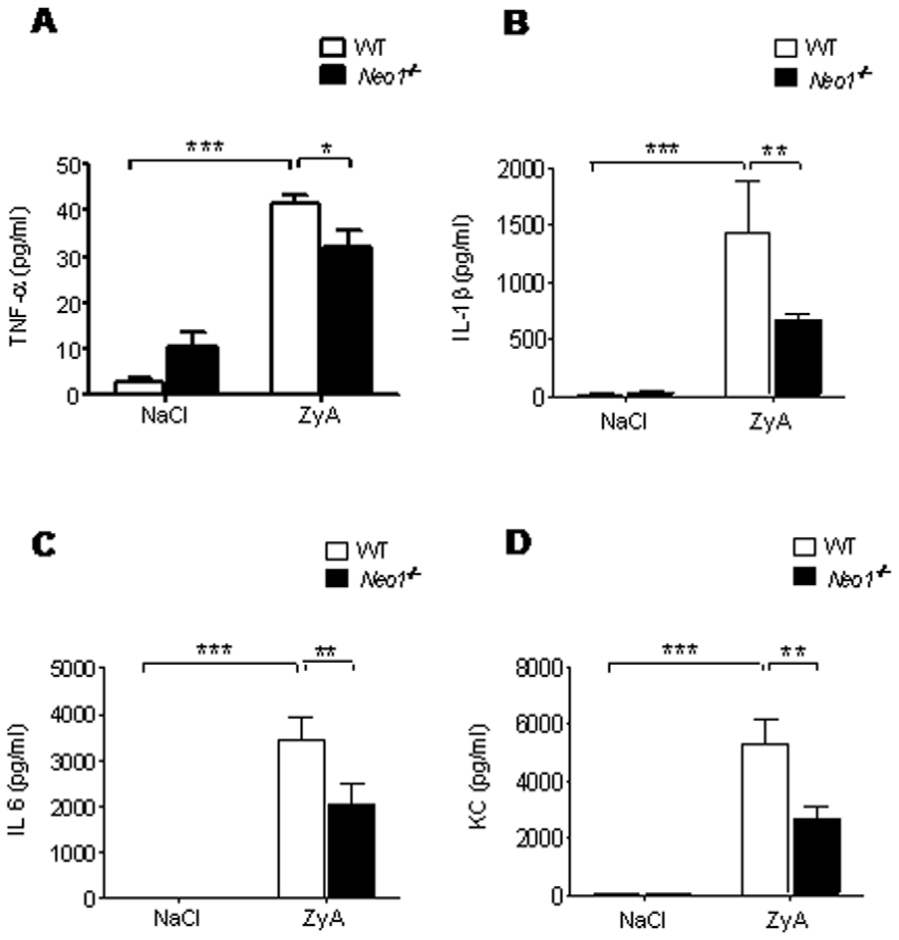
Neogenin increases inflammatory cytokine release *in-vivo*. *Neo1^−/−^* and *WT* animals were injected i.p. with NaCl or 1 mg of zymosan A (ZyA) and peritoneal lavage obtained after 8 hours **A**) TNF-α concentration in peritoneal lavage of *Neo1^−/−^* and WT animals **B**) IL-1β concentration in peritoneal lavage of *Neo1^−/−^* and WT animals **C**) IL-6 concentration in peritoneal lavage of *Neo1^−/−^* and WT animals **D**) KC concentration in peritoneal lavage of *Neo1^−/−^* and WT animals 8 hours following intraperitoneal NaCl or ZyA injection (Data are Mean ± SEM, n = 6 per group, *P<0.05, **P<0.01, ***P<0.001 as indicated).

We then proceeded to investigate the functional implications of neogenin during an acute inflammatory response using previously characterized *Neo1^−/−^* mice in a model of ZyA induced peritonitis. 8 hours following ZyA injection *Neo1^−/−^* mice demonstrated significantly reduced number of cells in the peritoneal lavage compared to WT animals (×10^6^, *Neo1^−/−^* 3.02±0.3 vs. WT 4.3±0.4, p<0.01; Figure 2A) This result was reflected when evaluating the MPO activity and the protein content within the peritoneal lavage (Figure 2B and C). To assess possible histological changes of the peritoneal cavity following the inflammatory response we next performed histological sections of the peritoneal cavity, the mesenterial fat and the cellular exudate. *Neo1^−/−^* mice demonstrated significantly reduced inflammatory changes and tissue destruction of the peritoneal and decreased infiltration of inflammatory cells into the mesenterial fat compared to WT animals (Figure 2D). Cytospin samples verified the reduced number of inflammatory cells in the peritoneal lavage of *Neo1^−/−^* mice compared to the WT animals. We also evaluated the level of inflammatory cytokines within the peritoneal lavage and found that *Neo1^−/−^* mice demonstrated significantly reduced levels of TNF-α (pg/ml, *Neo1^−/−^* 32±4 vs. WT 42±2, p<0.01), IL-1β (pg/ml, *Neo1^−/−^* 662±63 vs. WT 1435±451, p<0.05), IL-6 (pg/ml, *Neo1^−/−^* 2027±478 vs. WT 3445±489, p<0.05) and KC (pg/ml, *Neo1^−/−^* 2657±446 vs. WT 5270±853, p<0.05), compared to WT animals (Figure 3A–D).

### Functional inhibition of neogenin dampens acute inflammatory peritonitis

**Figure 4:**
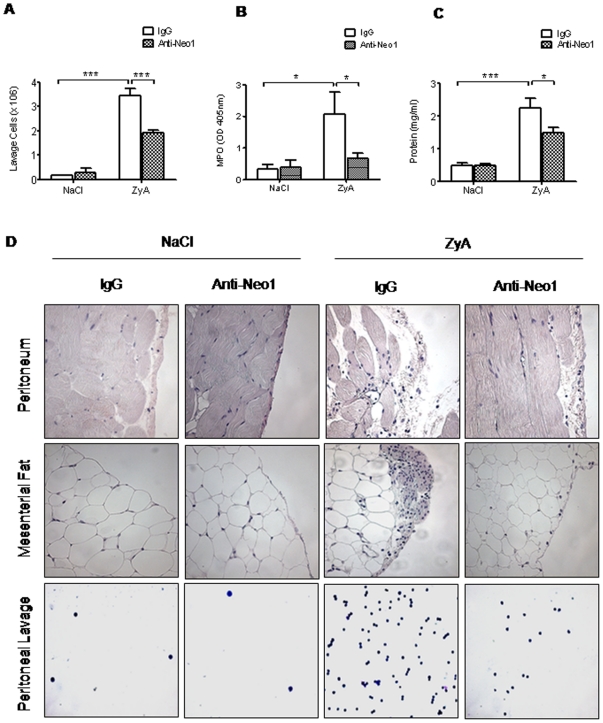
Functional inhibition of neogenin dampens acute inflammatory peritonitis. WT animals were injected i.p. with NaCl or 1 mg of zymosan A (ZyA) and subsequently i.v. with either IgG control or anti-Neogenin (Anti-Neo1) antibody (1 µg) and peritoneal lavage obtained after 8 hours **A**) Cell count in peritoneal lavage of *Neo1^−/−^* and WT animals **B**) Myeloperoxidase (MPO) activity in peritoneal lavage **C**) Protein content in peritoneal lavage of *Neo1^−/−^* and WT animals **D**) Representative histological analysis of the peritoneum, the mesenterial fat and cytospin samples of the peritoneal lavage in *Neo1^−/−^* and WT animals 8 hours following intraperitoneal NaCl or ZyA injection. Sections prepared with hematoxylin-eosin staining (Magnification ×400, insert ×1000; Data are Mean ± SEM, n = 6 per group, *P<0.05, **P<0.01, ***P<0.001 as indicated).

**Figure 5:**
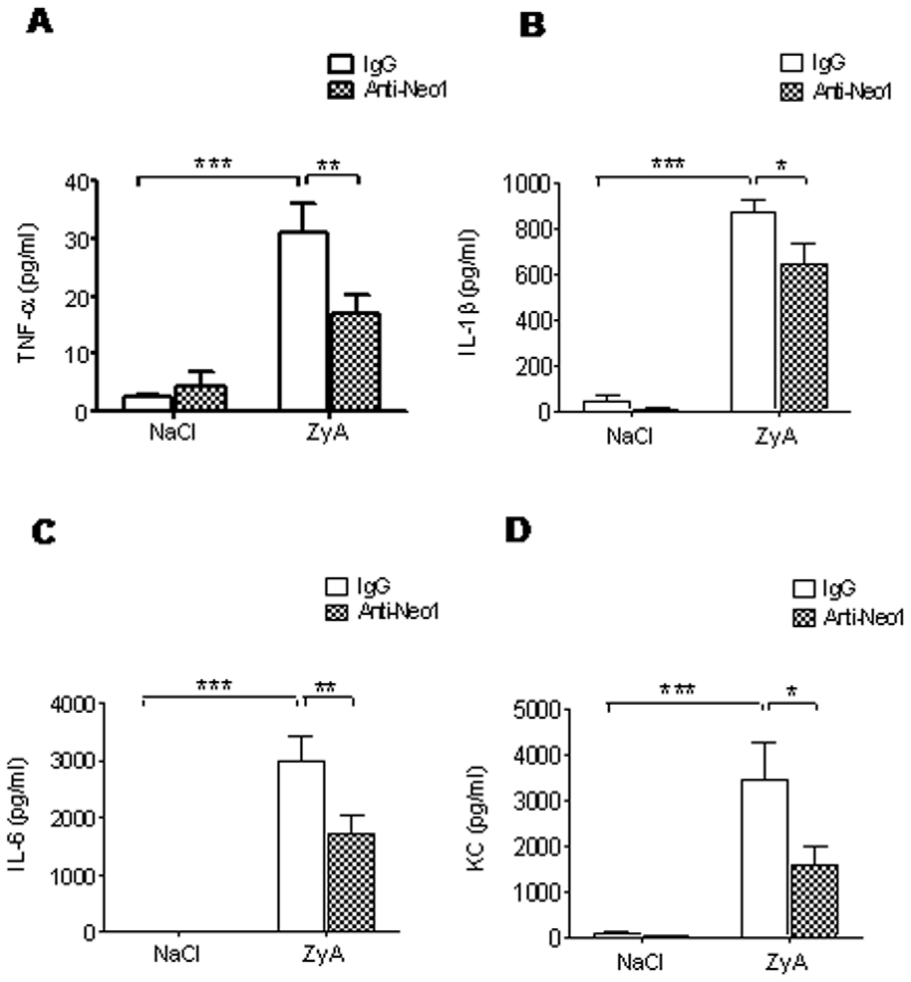
Functional inhibition of neogenin dampens release of inflammatory cytokines *in-vivo*. WT animals were injected i.p. with NaCl or 1 mg of zymosan A (ZyA) and subsequently i.v. with either IgG control or anti-Neogenin (Anti-Neo1) antibody (1 µg) and peritoneal lavage obtained after 8 hours **A**) TNF-α concentration in peritoneal lavage **B**) IL-1β concentration in peritoneal lavage **C**) IL-6 concentration in peritoneal lavage **D**) KC concentration in peritoneal lavage of WT animals injected with either vehicle or Anti-Neo1 (Data are Mean ± SEM, n = 6 per group, *P<0.05, **P<0.01, ***P<0.001 as indicated).

To further define the role of neogenin during acute inflammatory peritonitis we injected WT animals with functionally inhibiting neogenin antibody (anti-Neo1) and appropriate IgG isotype as control. Following this we found a significant reduction of the cell number within the peritoneal fluid of anti-Neo1 injected animals compared to IgG controls (×10^6^, anti-Neo1 1.90±0.13 vs. IgG 3.44±0.3, p<0.01; Figure 4A). This difference between groups was also present when evaluating the MPO activity and the protein content within the peritoneal lavage (Figure 4B and C). The evaluation of histological sections demonstrated significant attenuation of tissue destruction and infiltration of inflammatory cells into the mesenterial fat following anti-Neo1 injection (Figure 4D). To further strengthen this finding we evaluated cytokine levels within the peritoneal lavage and found a reduction of TNF-α (pg/ml, anti-Neo1 17±3 vs. IgG 31±5, p<0.01), IL-1β (pg/ml, anti-Neo1 643±87 vs. IgG 871±51, p<0.05), IL-6 (pg/ml, anti-Neo1 1725±326 vs. IgG 2988±425, p<0.05) and KC (pg/ml, anti-Neo1 1576±406 vs. IgG 3421±851, p<0.05) (Figure 5A–D).

### Haematopoetic neogenin is crucial for inflammatory control following peritonitis

Based on the above studies we aimed to identify whether tissue specific or haematopoetic neogenin expression would be responsible for the findings described above. For this purpose, we employed chimeric animals by transferring BM from WT to *Neo1* ***^−/−^*** mice and vice versa, with WT to WT and *Neo1* ***^−/−^*** to *Neo1* ***^−/−^*** transplanted animals as controls for non-specific effects caused by irradiation.

**Figure 6:**
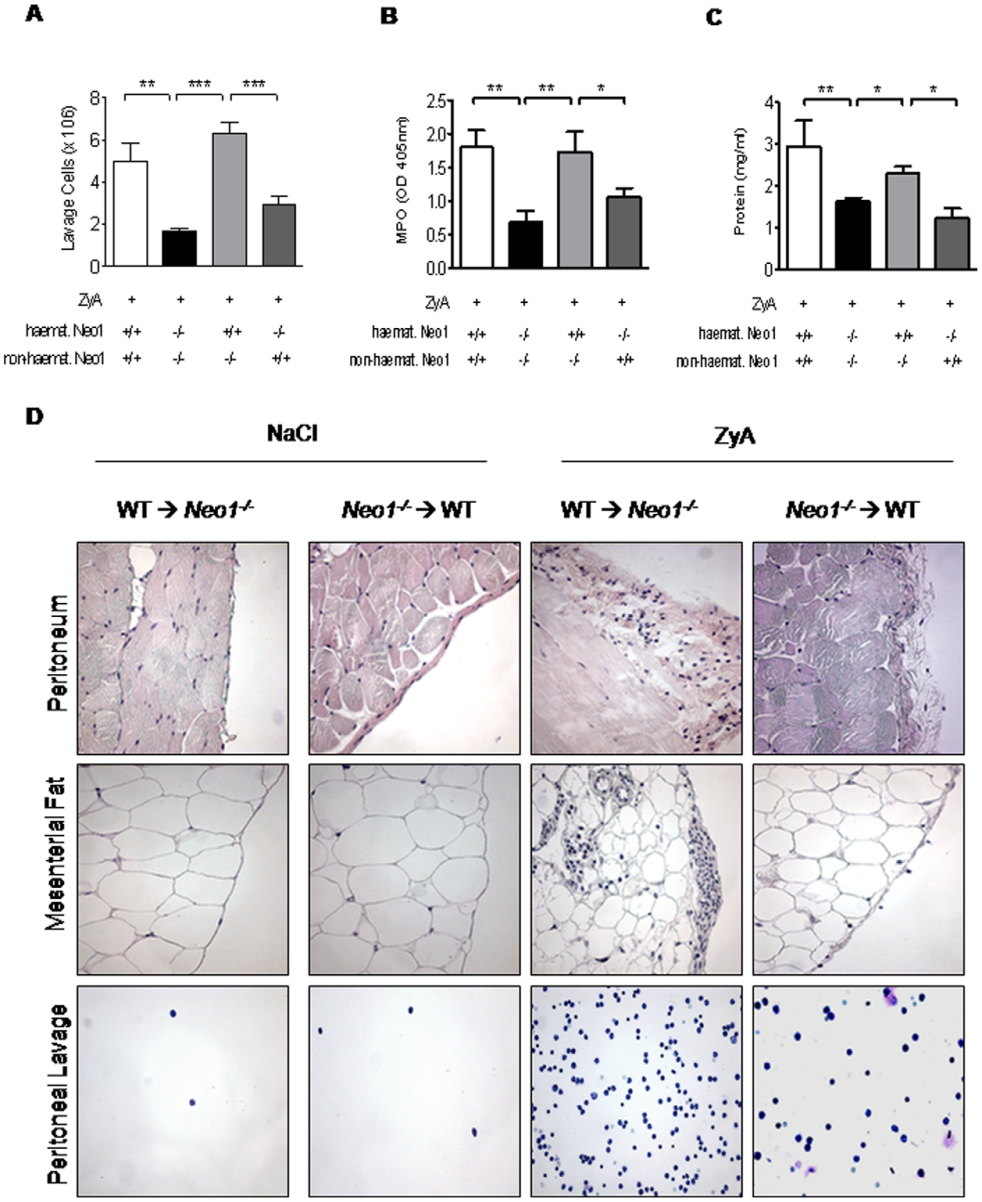
Loss of haematopoietic neogenin decreases acute peritoneal inflammation. Chimeric animals and controls were injected i.p. with zymosan A (ZyA) and peritoneal lavage obtained after 8 hours **A**) Cell count within the peritoneal fluid in chimeric animals and controls 8 h after exposure to ZyA **B**) Myeloperoxidase (MPO) activity in peritoneal lavage **C**) Protein content in peritoneal lavage of chimeric animals and controls **D**) Representative histological analysis of the peritoneum, the mesenterial fat and cytospin samples of the peritoneal lavage of chimeric animals and controls 8 hours following intraperitoneal ZyA injection. Sections prepared with hematoxylin-eosin staining (Magnification ×400, insert ×1000). (Data are Mean ± SEM, n = 6 per group, *P<0.05, **P<0.01, ***P<0.001 as indicated).

**Figure 7:**
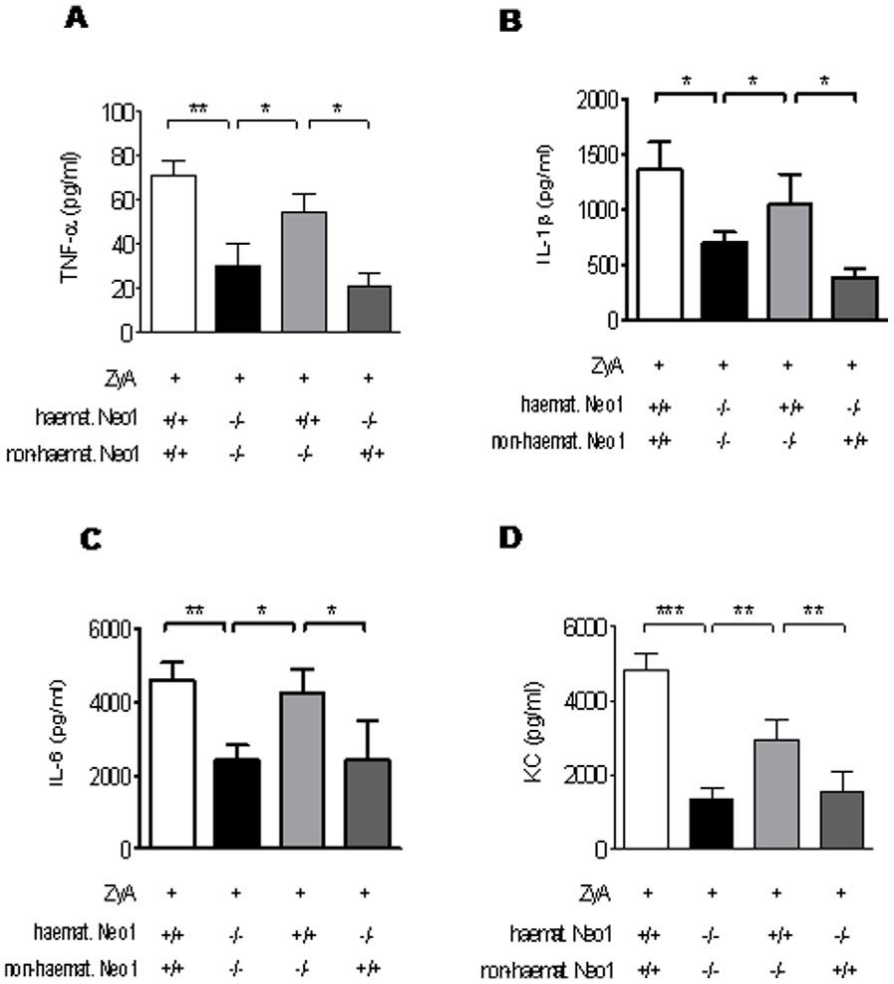
Loss of haematopoietic neogenin dampens cytokine release during acute inflammatory peritonitis. Chimeric animals and controls were injected i.p. with 1 mg of zymosan A (ZyA) and samples taken after 8 hours **A**) TNF-α concentration in peritoneal lavage **B**) IL-1β concentration in peritoneal lavage **C**) IL-6 concentration in peritoneal lavage **D**) KC concentration in peritoneal lavage of chimeric animals and controls injected with zymosan A (Data are Mean ± SEM, n = 6 per group, *P<0.05, **P<0.01, ***P<0.001 as indicated).

We then exposed these chimeric animals to the previously described model of ZyA induced peritonitis and assessed the inflammatory response as described above. Baseline characteristics following intraperitoneal NaCl injection did not differ between groups (Supplemental Figure S1 A–C). Bone marrow chimeric animals with haematopoetic neogenin repression (haemat. *Neo1^−/−^*) demonstrated a significantly decreased cell number in peritoneal fluid compared to the animals with haematopoetic neogenin expression (haemat. *Neo1^+/+^*) when exposed to ZyA (haemat. *Neo1* ***^−/−^*** 2.9±0.41, haemat. *Neo1^+/+^* 6.30±0.5, p<0.05; Figure 6A). This was confirmed when evaluating the MPO activity and the protein content within the peritoneal lavage (Figure 6B and C). Again, we performed histological evaluation of the inflammatory changes within the peritoneal cavity following i.p. NaCl or ZyA injection. The WT to WT and *Neo1* ***^−/−^*** to *Neo1* ***^−/−^*** transplanted animals reflected the findings of the WT or *Neo1* ***^−/−^*** animals (Supplemental Figure S2). When determining the histological changes in the peritoneal cavity of chimeric animals we found significantly decreased histological damage in the haemat. *Neo1* ***^−/−^*** animals compared to haemat. *Neo1^+/+^* animals (Figure 6D). The cytokine response within the peritoneal lavage did not differ between groups following i.p. NaCl injection (Supplemental Figure S3). When exposed to ZyA we found a reduction of TNF-α in haemat. *Neo1* ***^−/−^*** animals compared to haemat. *Neo1^+/+^* (Figure 7A). This finding was also present when determining the concentration of IL-1β, IL 6 and KC (Figure 7B to D). Taken together these studies suggest that the haematopoetic expression of neogenin significantly promotes an inflammatory response.

## Discussion

Recent work has demonstrated that the nervous and the immune system share basic biological principles. One of these parallels is the function of neuronal guidance proteins (NGP)s that were initially described in the context of axonal migration but were demonstrated to also serve as endogenous guidance cues for leukocyte migration. Neogenin is an important target receptor for NGPs during axonal growth and neuronal development yet its role during acute inflammation to date was not elucidated. We report here that neogenin is an important propagator of an acute inflammatory response during the initial stages of inflammatory peritonitis. *Neo1^−/−^* animals demonstrated significantly reduced recruitment of inflammatory cells into the peritoneal cavity and attenuated cytokine response following i.p ZyA injection. Studies using functional inhibition of neogenin through antibody confirmed these findings. Furthermore, in studies employing chimeric animals we were able to demonstrate that haematopoetic Neo1 expression is crucial for the observed findings. With these studies we identified neogenin, a neuronal guidance receptor that is involved into the propagation of axonal growth, to also be an important propagator of an acute inflammatory response.

Neogenin was initially described by Vielmetter et al. when searching for proteins involved into neurite extension and neuronal signaling [24]. Subsequent work has then demonstrated that neogenin is not only expressed during neuronal development but is also present in a variety of adult tissues. Keeling et al. demonstrated that neogenin holds important function for the differentiation of murine adult tissues and also demonstrated that neogenin is expressed in organs of key importance for an immune response such as the spleen, intestine and the thymus [25]. Further studies identified the role of neogenin during neurogenesis, during myogenic differentiation, during chondrogenesis and during angiogenesis confirming the important role of neogenin during cellular migration [17], [21], [22], [26], [27]. However, none of these studies described the expression of neogenin on immune competent cells. In a recent study investigating the role of the UNC5b receptor during renal ischemia-reperfusion the investigators were unable to demonstrate an expression of neogenin on murine leukocytes [28]. We report here a robust expression of neogenin in several murine organs and corroborate the previous findings demonstrating neogenin in adult tissues. In addition we found a robust expression of neogenin on CD45 positive cells which implicates neogenin to be present on immune competent cells. This implicates a possible functional role for neogenin during an acute inflammatory response. Our finding here confirms previous findings of our group demonstrating the expression of neogenin on human leukocytes [11].

The functional role of neogenin signaling is well described in the context of neuronal development. During axonal growth netrin-1 binds to neogenin which results in a chemoattractive signal, but when binding to the repulsive guidance molecule A (RGM-A) neogenin serves as a chemorepulsive receptor for neuronal migration [14], [15]. Recent work has found that basic biological principles, such as cellular guidance and migration are shared by the nervous and the immune system. This work led to the discovery that NGPs hold additional functional implications outside the CNS. For example, Slit-2 and Netrin-1 were reported to serve as stop signal for the migration of granulocytes, lymphocytes, monocytes and dendritic cells [6], [29],[6], [30]–[33]. First evidence that neogenin might be involved into the control of an acute inflammatory response came from our previous study describing the role of RGM-A during an acute inflammatory response. In this study we found that RGM-A reduces the chemotactic migration of neutrophils. We also found that RGM-A mitigates the extent of an acute inflammatory response in-vivo in dependence of the neogenin receptor [11]. Further evidence for the role of RGM-A was provided during chronic inflammatory changes in the nervous system in which RGM-A activates T-cells in a model of multiple sclerosis. In the study by Maramatsu et al. the authors define an activating role for RGM-A on T-cell mediated cytokine release within the CNS [5]. These studies described different aspects of the role of neogenin during inflammation and implicated to further define the role of neogenin during acute inflammation. We report here that neogenin propagates an acute inflammatory response at a later time point then RGM-A exerts its anti-inflammatory potential. Whether RGM-A is therefore a functional agonist or antagonist when binding to neogenin on immune cells in the context of acute inflammation will be an important task to further define in subsequent studies. Therefore further work will be needed to decipher the implications of netrin-1 or RGM-A binding to neogenin during an acute inflammatory response and the impact of this on an acute inflammatory response.

In conclusion we identified a previously uncharacterized function for neogenin outside the CNS. Our findings suggest that signaling through the neogenin receptor holds pro-inflammatory potential and intensifies inflammatory tissue changes. Furthermore, the findings in this study highlighted the importance of hematopoietic neogenin during acute peritoneal inflammation, and that a functional inhibition of neogenin dampens an acute inflammatory response. The identification of endogenous factors that influence infection and inflammation has important medical applications and may potentially be used to enhance regeneration and recovery following inflammatory changes in the future.

## Supporting Information

Figure S1 **Chimeric animals and controls exposed to intraperitoneal NaCl injection.** **A**) Cell count within the peritoneal fluid in chimeric animals and controls 8 hours following intraperitoneal NaCl injection **B**) Myeloperoxidase (MPO) activity in peritoneal lavage **C**) Protein content in peritoneal lavage of chimeric animals and controls 8 hours following intraperitoneal NaCl injection. (Data are Mean ± SEM, n = 6 per group).
(TIF)

Figure S2 **Histological assessment of chimeric animals and controls exposed to intraperitoneal NaCl injection.** Representative histological analysis of the peritoneum, the mesenterial fat and cytospin samples of the peritoneal lavage of chimeric animals and controls 8 hours following intraperitoneal NaCl injection. Sections prepared with hematoxylin-eosin staining (Magnification ×400, insert ×1000).
(TIF)

Figure S3 **Cytokine concentration in chimeric animals and controls exposed to intraperitoneal NaCl injection.** **A**) TNF-α concentration in peritoneal lavage **B**) IL-1β concentration in peritoneal lavage **C**) IL-6 concentration in peritoneal lavage **D**) IL-8 concentration in peritoneal lavage of chimeric animals and controls injected with zymosan A 8 hours following intraperitoneal NaCl injection (Data are Mean ± SEM, n = 6 per group).
(TIF)
