# Supplementary figures and images for: The Axonal Guidance Receptor Neogenin Promotes Acute Inflammation
Source: PLoS One. 2012 Mar 6;7(3):e32145. doi: 10.1371/journal.pone.0032145 (PMC3295751; doi:10.1371/journal.pone.0032145)

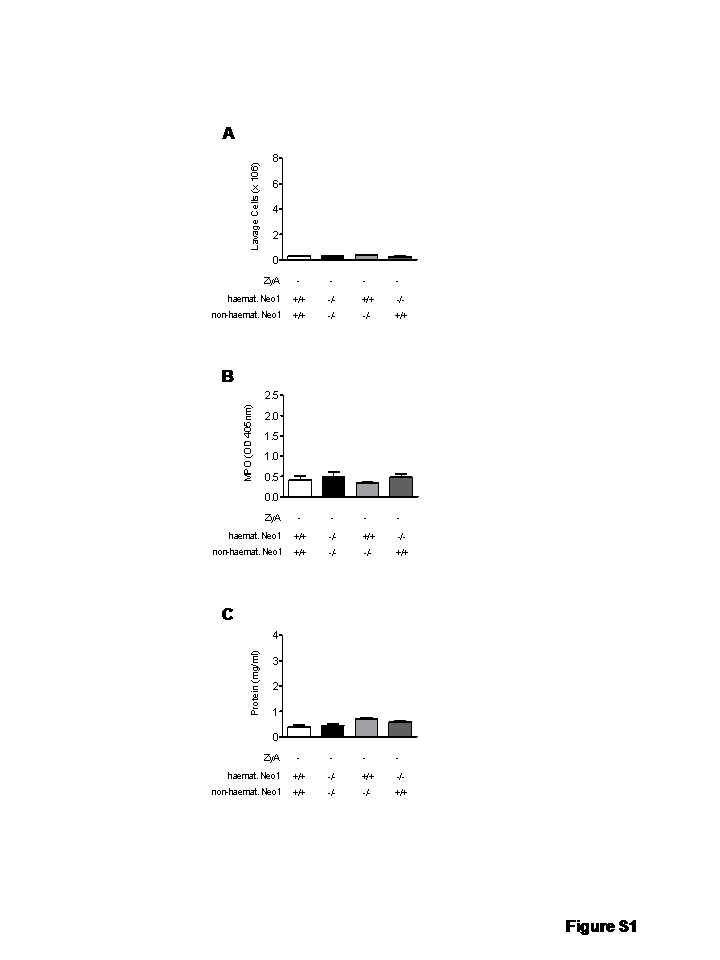

Supplement: Figure S1 — Chimeric animals and controls exposed to intraperitoneal NaCl injection. A) Cell count within the peritoneal fluid in chimeric animals and controls 8 hours following intraperitoneal NaCl injection B) Myeloperoxidase (MPO) activity in peritoneal lavage C) Protein content in peritoneal lavage of chimeric animals and controls 8 hours following intraperitoneal NaCl injection. (Data are Mean ± SEM, n = 6 per group). (TIF) [file pone.0032145.s001.tif]

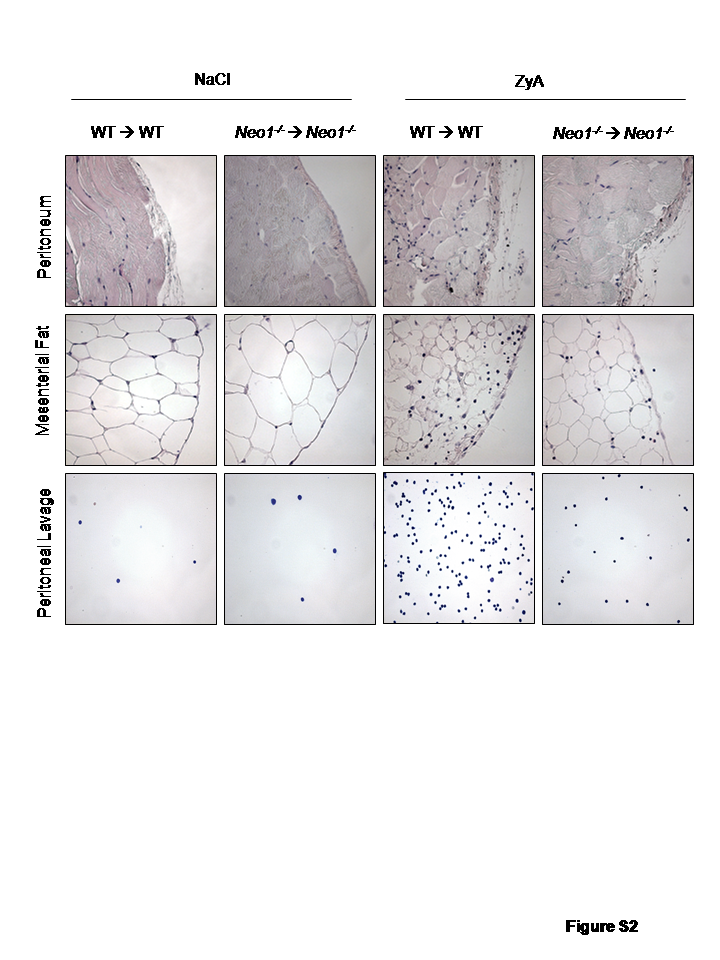

Supplement: Figure S2 — Histological assessment of chimeric animals and controls exposed to intraperitoneal NaCl injection. Representative histological analysis of the peritoneum, the mesenterial fat and cytospin samples of the peritoneal lavage of chimeric animals and controls 8 hours following intraperitoneal NaCl injection. Sections prepared with hematoxylin-eosin staining (Magnification ×400, insert ×1000). (TIF) [file pone.0032145.s002.tif]

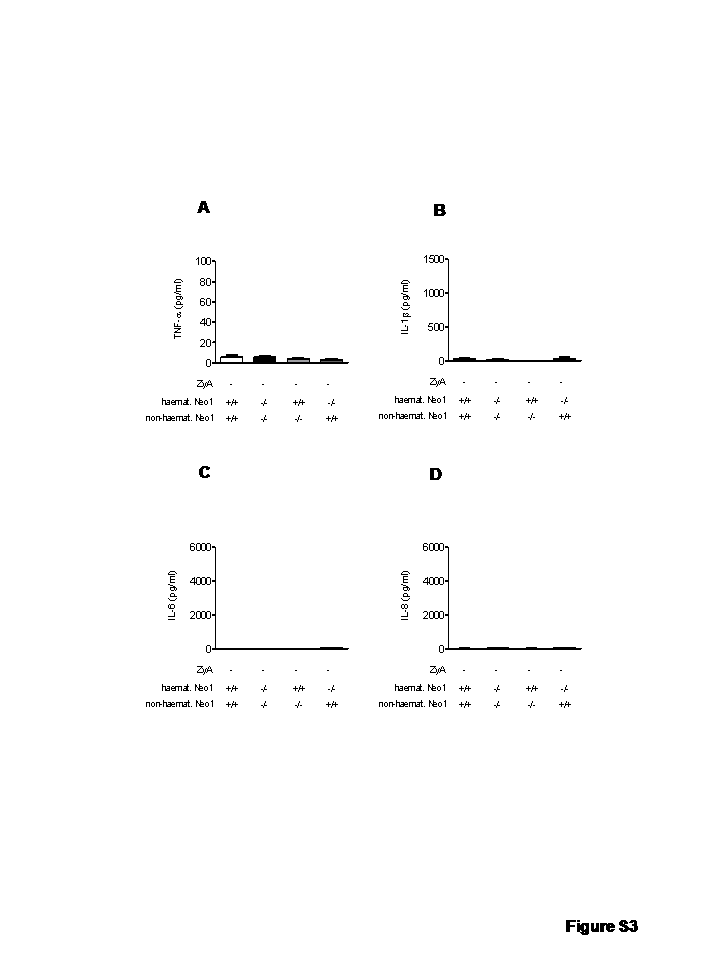

Supplement: Figure S3 — Cytokine concentration in chimeric animals and controls exposed to intraperitoneal NaCl injection. A) TNF-α concentration in peritoneal lavage B) IL-1β concentration in peritoneal lavage C) IL-6 concentration in peritoneal lavage D) IL-8 concentration in peritoneal lavage of chimeric animals and controls injected with zymosan A 8 hours following intraperitoneal NaCl injection (Data are Mean ± SEM, n = 6 per group). (TIF) [file pone.0032145.s003.tif]
